# Estimating Regional Spatial and Temporal Variability of PM_2.5_ Concentrations Using Satellite Data, Meteorology, and Land Use Information

**DOI:** 10.1289/ehp.0800123

**Published:** 2009-01-28

**Authors:** Yang Liu, Christopher J. Paciorek, Petros Koutrakis

**Affiliations:** 1 Department of Environmental Health and; 2 Department of Biostatistics, Harvard University, School of Public Health, Boston, Massachusetts, USA

**Keywords:** AOD, GAM, GASP, GOES, PM_2.5_, RUC, satellite aerosol remote sensing, spatial synoptic classification

## Abstract

**Background:**

Studies of chronic health effects due to exposures to particulate matter with aerodynamic diameters ≤ 2.5 μm (PM_2.5_) are often limited by sparse measurements. Satellite aerosol remote sensing data may be used to extend PM_2.5_ ground networks to cover a much larger area.

**Objectives:**

In this study we examined the benefits of using aerosol optical depth (AOD) retrieved by the Geostationary Operational Environmental Satellite (GOES) in conjunction with land use and meteorologic information to estimate ground-level PM_2.5_ concentrations.

**Methods:**

We developed a two-stage generalized additive model (GAM) for U.S. Environmental Protection Agency PM_2.5_ concentrations in a domain centered in Massachusetts. The AOD model represents conditions when AOD retrieval is successful; the non-AOD model represents conditions when AOD is missing in the domain.

**Results:**

The AOD model has a higher predicting power judged by adjusted *R*^2^ (0.79) than does the non-AOD model (0.48). The predicted PM_2.5_ concentrations by the AOD model are, on average, 0.8–0.9 μg/m^3^ higher than the non-AOD model predictions, with a more smooth spatial distribution, higher concentrations in rural areas, and the highest concentrations in areas other than major urban centers. Although AOD is a highly significant predictor of PM_2.5_, meteorologic parameters are major contributors to the better performance of the AOD model.

**Conclusions:**

GOES aerosol/smoke product (GASP) AOD is able to summarize a set of weather and land use conditions that stratify PM_2.5_ concentrations into two different spatial patterns. Even if land use regression models do not include AOD as a predictor variable, two separate models should be fitted to account for different PM_2.5_ spatial patterns related to AOD availability.

Environmental epidemiologic studies have established a robust association between chronic exposure to ambient-level fine particles [particulate matter (PM) with aerodynamic diameter ≤ 2.5 μm (PM_2.5_)] and adverse health effects such as mortality, ischemic heart disease, and lung cancer (Pope et al. 2002). Limited by data availability, large-scale chronic health effects studies of PM_2.5_ have used city-average concentrations measured at central ground monitors as representative of population exposures (e.g., Dockery et al. 1993). Using city-average exposures ignores within-city exposure contrasts and may underestimate the magnitude of the association between PM_2.5_ pollution and health outcomes. Miller et al. (2007) studied the association between long-term PM_2.5_ exposure and the incidence of cardiovascular diseases among women and reported that simultaneously estimated between-city effects and within-city effects are comparable. Jerrett et al. (2005) applied kriging techniques to study the association between within-city PM_2.5_ exposure gradients and mortality risks and found a substantially larger effect than previously reported using only city-average exposures.

With broad spatial coverage, satellite data can potentially expand ground monitoring networks into rural and suburban areas. In contrast to ground-level PM_2.5_ measurements, satellite sensors provide aerosol optical depth (AOD), a quantitative measure of PM abundance in the atmospheric column. Except for long-range dust or pollution transport events, AOD is dominated by near-surface emissions sources (Seinfeld and Pandis 1998). AOD retrieved at visible wavelengths is most sensitive to PM 0.1–2 μm (Kahn et al. 1998) and is not affected by gaseous copollutants. Unlike land use parameters, AOD provides a direct, albeit noisy, measurement of fine PM loading over an area rather than a surrogate of emission sources. Recent studies have established quantitative relationships between AOD and PM_2.5_ using linear regression models that include meteorologic parameters as covariates (Liu et al. 2005, 2007; van Donkelaar et al. 2006). Pelletier et al. (2007) studied the association between PM_10_ (PM ≤ 10 μm in aerodynamic diameter) concentrations and ground-based AOD measurements in Lille, France. Their model with smooth meteorologic terms performed significantly better than a linear regression model with AOD as the only predictor. Because they collected data from one site, their model could not represent the effect of location on the association between PM_10_ and meteorology. This resulted in substantial performance deterioration when they tested the model at another location. To our knowledge, no applications of satellite remote sensing data in spatial modeling of regional PM_2.5_ concentrations have been reported. Our work assesses the benefits of combining satellite data, meteorology, and land use information in a spatial statistical model to predict the spatial and temporal variability in daily PM_2.5_ concentrations at regional scale (i.e., the AOD model). Because AOD values are available only under cloud-free conditions, we also developed a similar statistical model using data collected where AOD is not available (i.e., the non-AOD model). Finally, to examine the different spatial patterns of PM_2.5_ concentrations divided by AOD availability, we made predictions where AOD was available using the fitted non-AOD model and compared these with predictions made by the AOD model.

## Materials and Methods

### U.S. Environmental Protection Agency PM_2.5_ concentrations

**Figure 1:**
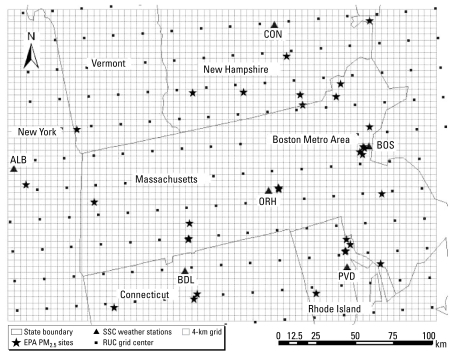
Study domain and the distributions of U.S. EPA PM_2.5_ sites, SSC weather stations, and RUC grid centroids. Thick lines show state borders, and the thin lines show the 4-km modeling grid.

The study domain is a 200 × 250 km^2^ rectangle covering Massachusetts (except for Cape Cod) and part of surrounding states (Figure 1). Among the 32 PM_2.5_ monitoring sites of the U.S. Environmental Protection Agency (EPA) compliance network operated by state and local government agencies on an every-3-day or every-6-day schedule in this area, 4 are in rural locations, 8 in suburban areas, and 20 in urban centers. We obtained daily-average PM_2.5_ concentrations by gravimetric methods from U.S. EPA (2007). We averaged measurements from collocated monitors for model fitting. We also used these measurements for estimating uncertainty attributable to instrument errors. The study period was from April 2003 to June 2005 (801 days), during which all sites operated for at least 6 months. We created a 4-km resolution grid for data spatial alignment and full-domain prediction. We selected this spatial resolution to match the nominal resolution of satellite data.

### Geostationary Operational Environmental Satellite aerosol/smoke product AOD

The Geostationary Operational Environmental Satellite (GOES) is the major weather satellite operated by the National Oceanic and Atmospheric Administration (NOAA). GOES aerosol/smoke product (GASP) AOD is estimated using lookup tables generated by a radiative-transfer model and surface reflectances calculated using clear-sky composite background images (Knapp et al. 2002). The satellite’s geostationary orbit allows AOD retrievals at 30-min frequencies between sunrise and sunset in cloud-free conditions. Regression on binned GASP AOD values against the ground truth at 10 northeastern U.S. and Canadian sites found a correlation coefficient of 0.79 (Prados et al. 2007). Paciorek et al. (2008) found that daily correlations between AOD and PM_2.5_ over time at fixed locations are reasonably high in the eastern United States except in winter. We obtained AOD data from the GASP team at NOAA National Environmental Satellite, Data, and Information Service. Data screening criteria for outliers and residual cloud contamination followed Prados et al. (2007) and Kondragunta et al. (2008). We averaged AOD measurements corresponding to 1000–1500 hours local time to generate daily AOD estimates. The median number of AOD retrievals per day is 4; therefore, daily average GASP AOD is expected to create a better match to daily PM_2.5_ measurements compared with the snapshots taken by polar-orbiting satellite sensors.

### Meteorologic parameters

The relationship between AOD and PM_2.5_ concentrations can be modified by meteorologic parameters such as mixing height, relative humidity (RH), air temperature, and wind speed (Koelemeijer et al. 2006; Liu et al. 2005, 2007; Pelletier et al. 2007; van Donkelaar et al. 2006). We generated the meteorologic fields in the present analysis by the rapid update cycle (RUC) model, a numerical weather forecast system developed by the Earth System Research Laboratory RUC development group at NOAA (Benjamin et al. 2004). RUC data integrate observations from various surface networks, commercial aircraft, and satellites and are available at 20-km spatial resolution (Department of Energy Office of Science 2007). The 1-hr frequency of the RUC model enabled us to average various meteorologic parameters at the corresponding averaging time window of GASP AOD. The black squares in Figure 1 show the centroids of each 20-km RUC pixel.

### Spatial synoptic classification

Synoptic weather types have been used as an alternative to individual meteorologic parameters in modifying the association between pollution and health outcomes (Pope and Kalkstein 1996). The spatial synoptic classification (SSC) is a semiautomatic classification scheme calculated based on daily observations of temperature, dew point, wind, pressure, and cloud cover at an individual station. A detailed explanation of SSC weather types has been previously published (Sheridan 2002). We obtained SSC data from six weather stations in or surrounding the modeling domain (solid triangles in Figure 1) from Kent State University (Sheridan 2007) and used these to create a three-level (i.e., dry tropical, moist tropical, and otherwise) categorical variable based on preliminary analysis.

### Land use parameters

Various traffic and land use indicators such as distance to road, road length, traffic volume, land use type, population information, and altitude have been used to predict air pollution concentrations at intraurban scale, and no conclusion has been drawn on which are best predictors of air pollution levels (Brauer et al. 2003). We compiled elevation and road length data for three classes of roads using raw data from ESRI StreetMap USA (Environmental Systems Research Institute, Inc., Redlands, CA), land use types from the U.S. Geological Survey’s National Land Cover Database 2001 (Multi-resolution Land Characteristics Consortium 2007), and population density from U.S. 2000 Census data in each 4-km grid cell. In Massachusetts, the StreetMap data of major roadways are usually within approximately 30 m of the very accurate Massachusetts Highway Department data (Melly S, personal communication).

### Spatial alignment of data

To develop our spatial models, we matched all parameters to the 4-km grid. We report AOD data at the centroids of roughly 6.5 km × 2.4 km rectangular GOES pixels. To assign AOD values to the regular 4-km modeling grid, we created a network of Thiessen polygons in ArcGIS (version 9.1; ESRI), each of which is a rectangle with an area of 16 km^2^. We calculated the AOD value for each 4-km grid cell as the area-weighted average of the AOD values of the Thiessen polygons intersecting with this cell. AOD values at each U.S. EPA site are determined according to the grid cell in which the site falls. We determined the SSC types and RUC meteorologic parameters at each grid cell as well as at each U.S. EPA site by the nearest weather station (for SSC) or the nearest RUC pixel centroid.

### Construction of the AOD and non-AOD data sets

Unlike meteorologic and land use data with near complete coverage, AOD values are often missing because of cloud cover, high surface reflectance (e.g., snow cover), or retrieval errors. Including GASP AOD as a predictor in the model limits the spatial and temporal coverage of predicted daily PM_2.5_ concentrations. To evaluate the benefits of including GASP AOD data, we developed two models separately. The AOD model data set (2,570 site-days) includes those days when GASP AOD data can be matched to EPA PM_2.5_ measurements. The non-AOD model data set (7,009 site-days) includes those site-days when GASP AOD data are missing (i.e., PM_2.5_ observations not matched to AOD). The statistical model fitted with this data set excludes AOD (the non-AOD model), and is designed to evaluate the capability of meteorologic and land use information as predictors of PM_2.5_ levels when AOD is missing (Gryparis et al. 2007). The AOD domain data set for prediction includes all the grid cells and days when AOD is available during the study period as other variables have almost complete spatial and temporal coverage (1,023,112 grid-days), and the non-AOD domain data set includes the rest of the grid cells and days (1,809,622 grid-days). The AOD and non-AOD domain data sets together have complete spatial and temporal coverage in the domain.

### Model structure and prediction in the domain

We modeled the temporal and spatial variabilities in PM_2.5_ concentrations separately using two-stage generalized additive models (GAMs) (Hastie and Tibshirani 1990). We assumed normally distributed, homoskedastic model residuals at both stages. Both the AOD model and the non-AOD model share the common structure expressed in Equations 1–3 except that AOD is not included in the non-AOD model, and the two models are fitted with different data sets.

Stage 1 of the GAM aims to explain the temporal variability in PM_2.5_ concentrations in the modeling domain:


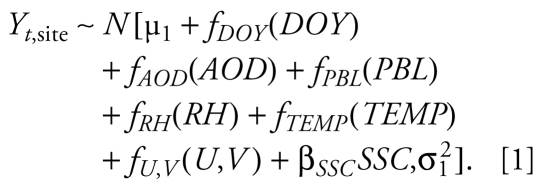


*Y_t_*_,site_ is the daily PM_2.5_ concentration at a given site. All the covariates (right-hand side of Equation 1) are averaged spatially and therefore vary only with time, except for *SSC*, because averaging the discrete SSC levels is meaningless. μ_1_ is the model intercept, *f_DOY_*(*DOY*) is a smooth regression term for day of year (DOY); *f_AOD_*(*AOD*) is the smooth regression term describing the association between domain-average AOD and *Y_t_*_,site_; and *f_PBL_*(*PBL*), *f_RH_*(*RH*), and *f_TEMP_*(*TEMP*) are smooth regression terms describing the impact of domain-averaged planetary boundary–layer height (PBL), RH, and surface air temperature (TEMP) on the AOD–PM_2.5_ association, respectively. Following Pelletier et al. (2007), we decompose the wind vector into the *U* component (west-to-east) and *V* component (south-to-north) instead of a scalar wind speed and wind direction bounded between 0 and 360 degrees. *f_U_*_,_*_V_*(*U*,*V*) is a two-dimensional smooth surface describing the impact of wind speed and direction on the AOD-PM_2.5_ association. SSC is modeled as a three-level categorical variable because of its discrete values.

Stage 2 of the GAM aims to explain the spatial variability in PM_2.5_ concentrations in the modeling domain:


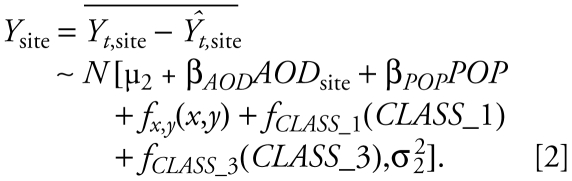


All the covariates in Equation 2 are averaged over the entire modeling period and therefore vary with only space. *Y*_site_ is calculated by averaging the residual PM_2.5_ concentrations from Equation 1 (i.e., *Y_t_*_,site_ − *Ŷ_t_*_,site_) at each site, so it contains only the spatial component of the variability in PM_2.5_ concentrations. μ_2_ is the model intercept, *AOD*_site_ is the average GASP AOD at a given site, and *POP* is population density at a given site. Both site-average AOD and population density are modeled as linear terms based on preliminary analysis. *f_x_*_,_*_y_*(*x*,*y*) is the pure spatial smooth surface reflecting the potential impact of location, represented by site coordinates, on the AOD–PM_2.5_ association. *f_CLASS_*__1_(*CLASS*_1) and *f_CLASS_*__3_(*CLASS*_3) are the smooth regression terms for class 1 (limited-access or interstate highways) and class 3 (secondary and connecting roads, state, and county highways) road lengths, respectively, in the grid cell in which a given site falls.

We considered a third stage to capture space–time interaction. However, preliminary analysis showed that the covariates that vary in space and time (i.e., AOD and weather variables) could not predict time-varying spatial PM_2.5_ surfaces in this domain. A more sophisticated model would account for space–time interaction using a statistical space–time covariance structure. However, given the greatly increased computational burden, we chose the simpler specification above, because a more sophisticated model structure would contribute little to our primary focus on the use of AOD for predicting PM_2.5_. Furthermore, having a space–time covariance in each model is difficult to interpret conceptually, given that the site-days at nearby locations and short time lags can appear in both data sets.

We fitted the AOD and non-AOD models with the gam() function in the mgcv package in R (Wood 2006). We represented the one-dimensional smooth terms such as *f_AOD_*(*AOD*), *f_PBL_*(*PBL*), *f_RH_*(*RH*), *f_TEMP_*(*TEMP*), *f_CLASS_*__1_(*CLASS*_1), and *f_CLASS_*__3_(*CLASS*_3) by penalized cubic splines for computational efficiency, which is particularly important in the full-domain prediction. Using the thin-plate spline basis yields very similar results. We represented the two-dimensional smooth terms such as *f_U_*_,_*_V_*(*U*,*V*) and *f_x_*_,_*_y_*(*x*,*y*) using penalized thin-plate splines. For all the spline terms, we estimated the amount of smoothing using generalized cross-validation (CV) (Wood 2006) as implemented by the gam() function. We limited the number of knots as an additional constraint to avoid potential overfitting. The final GAMs presented in this article are determined such that all the covariates are significant at α = 0.05 level, the model adjusted *R*^2^ is among the highest of all the models, and the correlations between covariates are among the lowest. We also considered other meteorologic parameters and land use types than those included in the final models, but they were not significant. We obtained the final prediction of PM_2.5_ concentration by summing the fitted values of Equations 1 and 2:


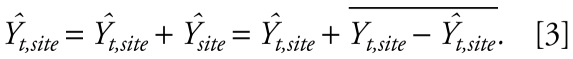


### Model validation

We validated our model using CV techniques, which test for potential overfitting; that is, the model could fit the data better at EPA sites than at the rest of the study area. In particular, after deciding which of the predictors to include in the final model using the whole data set, we sequentially retained data from one site as the testing data set, fitted the model to the remaining data, and then made predictions of daily PM_2.5_ concentrations at the testing site. We calculated prediction errors by subtracting retained observations from the model predictions. We estimated the model prediction precision by taking the square root of the mean squared prediction errors (RMSPE) (Yanosky et al. 2008). On completion of model development, we used both the AOD and the non-AOD models to predict daily PM_2.5_ concentrations in the domain using the corresponding domain data set.

## Results

### Descriptive statistics

**Figure 2:**
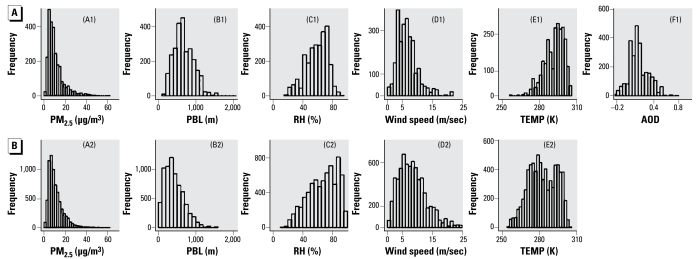
Summary statistics of major variables for the AOD model (*A*) and non-AOD model (*B*): daily PM_2.5_ concentrations (A1 and A2), domain-average PBL-layer height (B1 and B2), lower troposphere RH (C1 and C2) and wind speed (D1 and D2), surface temperature (E1 and E2), and GASP AOD (F1).

Figure 2 shows the histograms of major predictor variables in the two model data sets. Successful AOD retrievals require cloud-free conditions and low surface reflectance (i.e., no snow or ice on the ground), which tend to be associated with deep boundary layers, low RH, low wind speed, and high air temperature. The days without AOD retrievals are associated with the opposite weather pattern. The distributions of PM_2.5_ concentrations, however, were similar in the two data sets (plots A1 and A2). The overall mean PM_2.5_ concentration was 10.7 μg/m^3^ for the AOD data set and 10.8 μg/m^3^ for the non-AOD data set. In both data sets, median PM_2.5_ concentration was highest in the summer and lowest in the winter. Median AOD was highest in the summer (0.17) and lowest in the winter (0.09). Negative AOD values caused by errors in surface reflectivity estimation are included because they provide useful information on low AOD situations (Paciorek et al. 2008). The histograms and summary statistics of the two model data sets are highly comparable with their corresponding domain data sets, except that both domain data sets have more extreme values because of larger sample sizes.

### Model fitting and residual spatial autocorrelation

**Figure 3:**
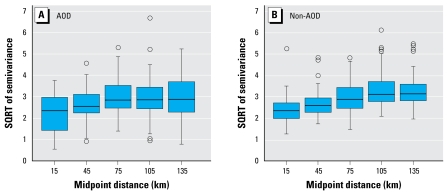
Residual spatial semivariance plots for the AOD model (*A*) and non-AOD model (*B*), with each point in a given box plot representing the square root (SQRT) of one-half of the average squared difference (of observations co-occurring in time) between two sites. Pairs of sites are binned based on the distance between them and only pairs with at least 10 co-occurring observations are included. Error bars indicate 95% confidence intervals; circles indicate outliers.

Table 1 summarizes model-fitting results for the AOD and non-AOD models. All the predictors listed in Table 1 are statistically significant at the α = 0.05 level. As indicated by adjusted *R*^2^ values, stages 1 and 2 of the AOD model explain 77% and 73% of the temporal and spatial variability in PM_2.5_, respectively. A linear regression between fitted (Equation 3) and observed PM_2.5_ concentrations yielded an adjusted *R*^2^ of 0.79 (correlation coefficient *r* = 0.89). Judged by adjusted *R*^2^ values, the spatial variability in PM_2.5_ captured by stage 2 contributes only 3% to total captured variability in PM_2.5_. Stages 1 and 2 of the non-AOD model explain 43% and 81% of the temporal and spatial variability in PM_2.5_, respectively. A linear regression between fitted and observed PM_2.5_ concentrations yielded an adjusted *R*^2^ of 0.48 (*r* = 0.70). Stage 2 of the non-AOD model contributes 10% to the total captured variability in PM_2.5_ concentrations. Semivariograms of the model residuals show some evidence of residual spatial autocorrelation in both models (Figure 3), which is caused by time-varying spatial variability not captured in our two-stage time plus space approach.

### Model validation and prediction errors

**Figure 4:**
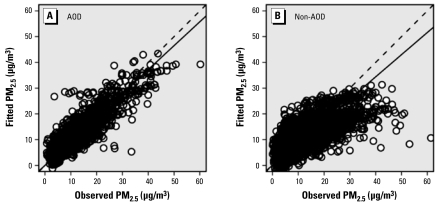
Scatterplots of CV predictions of daily PM_2.5_ concentrations versus U.S. EPA observations by the AOD model (*A*) and the non-AOD model (*B*). The solid line represents simple linear regression results with intercept excluded. The 1:1 line is displayed as a dashed line for reference. (*A*) fitted = 0.93 × observed; (*B*) fitted = 0.86 × observed.

For the AOD model, the linear CV *R*^2^, calculated from simple linear regression between fitted and observed PM_2.5_ concentrations ranged from 0.66 to 0.90 for each site with at least 20 data records. Table 1 shows that the overall linear CV *R*^2^ is 0.78, comparable with the model adjusted *R*^2^ of 0.79. For the non-AOD model, the linear CV *R*^2^ ranged more widely, from 0.31 to 0.74. The overall linear CV *R*^2^ is 0.46, comparable with the model adjusted *R*^2^ of 0.48. Overfitting is unlikely a serious issue in both models, although the AOD model performed more consistently compared with the non-AOD model. Although the mean prediction error is very small in both models (0.03 μg/m^3^ and 0.06 μg/m^3^, respectively), Figure 4 shows that predictions from both models are biased low at high concentration levels based on linear regressions between fitted and observed PM_2.5_ concentrations with intercepts forced through the origin (7% negative bias for the AOD model, and 14% negative bias for the non-AOD model). As a measure of prediction precision, the RMSPE is 3.5 μg/m^3^ for the AOD model and 5.0 μg/m^3^ for the non-AOD model. We use the fitted non-AOD model to predict the AOD site-days and found an adjusted *R*^2^ of 0.67 (CV *R*^2^ = 0.66) and a negative bias of 11% in CV model predictions. This is not fitting the AOD model excluding AOD to assess the additional effect of AOD on prediction. The lower adjusted *R*^2^ indicates that the non-AOD model does not represent the AOD model data set as well as the AOD model because the PM_2.5_ spatial patterns in the AOD model data set are different from those of the non-AOD model data set.

The CV *R*^2^ values suggest that 22% and 52% of the variability in PM_2.5_ concentrations are not explained by the AOD and the non-AOD models, respectively. In addition to unexplained space–time variability and unaccounted for variance when comparing areal predictions with point measurements, an additional source of variability in the observations is PM_2.5_ measurement error, which can be estimated by comparing collocated observations. There are 1,496 pairs of observations from 11 sites with collocated monitors, and the number of observations at each site ranges from 42 to 314. The overall median relative difference, calculated as median [2 × (PM_2.5__monitor_1 − PM_2.5__monitor_2)/(PM_2.5__monitor_1 + PM_2.5__monitor_2)], is 6.1%, and it ranges from 3.4% to 14.2% at different sites.

### Prediction of PM_2.5_ concentrations in the domain

We used both the AOD and non-AOD models to predict daily PM_2.5_ concentrations in the domain using the corresponding domain data sets. In the AOD domain data set, the total number of days with AOD data in each 4-km grid cell ranges from 134 to 326, with an average of 241 days, which corresponds to 17% to 41% temporal coverage, with an average of 30% coverage. Vermont and northwestern Massachusetts, with the most mountainous terrain in the domain, have the least coverage, whereas the coastal regions of Rhode Island and eastern Connecticut have the most coverage.

**Figure 5:**
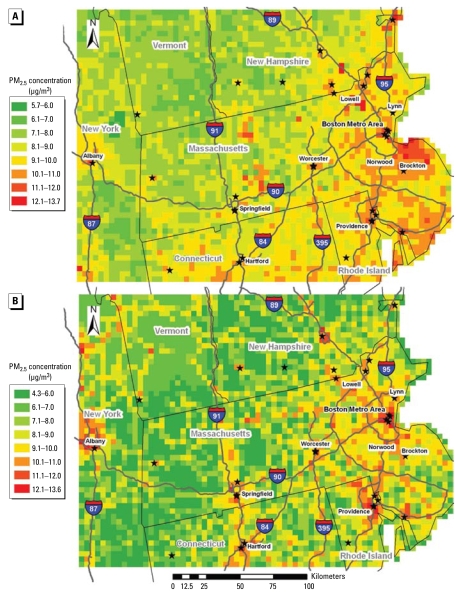
Mean PM_2.5_ concentrations during the entire modeling period predicted by the AOD model (*A*) and the non-AOD model (*B*). Stars represent the U.S. EPA monitoring sites. Urban areas with a population of > 100,000 (based on 2000 census data) and major interstate highways are also labeled (data from ESRI StreetMap USA).

Figure 5 shows the spatial pattern of predicted PM_2.5_ concentrations averaged over the entire period by both the AOD and the non-AOD models. For the AOD model predictions, mean predicted PM_2.5_ concentrations range from 5.7 μg/m^3^ near the border of New York and Vermont to 13.7 μg/m^3^ in Lowell, Massachusetts. As expected, predicted PM_2.5_ concentrations are higher in urban areas such as Boston, Massachusetts; Providence, Rhode Island; Hartford, Connecticut; and Albany, New York, compared with rural areas such as Vermont, western Massachusetts, and southwestern New Hampshire. However, high PM_2.5_ levels show a regional pattern, and the highest pollution levels are seen in the other areas of eastern Massachusetts instead of downtown Boston. In addition, grid cells along major highways (e.g., Highways 95, 89, and 395) tend to have higher PM_2.5_ concentrations, perhaps because these cells are also densely populated. The non-AOD model predictions have a similar range (from 4.3 μg/m^3^ in western Connecticut to 13.6 μg/m^3^ at downtown Boston). The predicted PM_2.5_ levels by the non-AOD model are comparable with the AOD model in major urban centers but significantly lower in rural areas. In addition, the high pollution levels are more isolated, with the highest concentrations all in downtown areas of Boston; Springfield, Massachusetts; Providence; and Hartford.

### Predictions at AOD grid-days using non-AOD model

**Figure 6:**
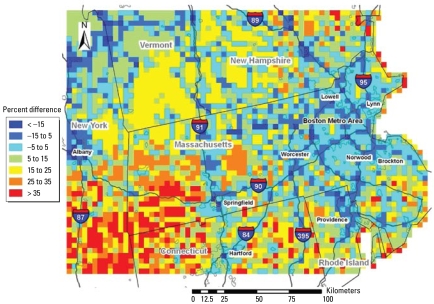
Differences between PM_2.5_ concentrations predicted by the AOD model (for grid-days with AOD available) and the non-AOD model (for grid-days with AOD not available) averaged over the days in the modeling period. Urban areas with a population of 100,000 or more (based on 2000 census data) and major interstate highways are also labeled (data from ESRI StreetMap USA).

To visualize the different PM_2.5_ spatial patterns separated by AOD availability, we used the fitted non-AOD model to predict daily PM_2.5_ concentrations on AOD grid-days in the entire domain. We compared this set of predictions, which have identical spatial and temporal coverage, with the AOD model predictions. The AOD model predictions are, on average, 0.8–0.9 μg/m^3^ higher than non-AOD model predictions. Figure 6 shows how the model predictions differ spatially. The relative differences between the non-AOD and the AOD model predictions, calculated as 2 × (AOD model prediction − non-AOD model prediction)/(AOD model prediction + non-AOD model prediction), vary substantially by region and land use type. Along major highways and in downtown areas, AOD model predictions are 5–15% lower than non-AOD model predictions, whereas predictions from the two models are comparable in suburban and urban areas. In rural areas, especially in the southwest of the domain, AOD model predictions are 25–50% higher than the non-AOD model predictions. In southern Vermont, AOD model predictions are 15–25% higher than non-AOD model predictions partially because annual mean AOD model predictions are biased high because of the lack of winter values.

## Discussion and Conclusions

The AOD model has a few important differences from the non-AOD model. First, the agreement between fitted PM_2.5_ concentrations and EPA observations is much better for the AOD model than for the non-AOD model, suggesting that PM_2.5_ is easier to predict when AOD is available. Second, stage 2 of the AOD model contributes only 3% to total captured variability in PM_2.5_ as judged by adjusted *R*^2^ values, whereas stage 2 of the non-AOD model contributes 10% to the total captured variability in PM_2.5_ concentrations. The two-stage GAMs in the present analysis are designed to capture the temporal and spatial variabilities in PM_2.5_ concentrations separately. Because the temporal variability dominates overall PM_2.5_ variability, it is not surprising to see that stage 1 of the GAMs largely determines the overall model performance. Finally, the AOD model predicts distinctly different spatial patterns of PM_2.5_ concentrations compared with the non-AOD model.

Figure 2 indicates that the success of AOD retrieval, determined by cloud cover, weather, and surface conditions, is systematically related to weather conditions. When AOD retrieval is successful (i.e., cloud free, higher temperature, no snow/ice on the ground), the total variability of PM_2.5_ concentrations is dominated by its temporal component, and concentration levels tend to be spatially smooth. Under such conditions, AOD, meteorologic parameters, and land use information are all effective predictors of PM_2.5_ concentrations in the AOD model. When AOD is missing because of cloud cover, high surface reflectance, or other reasons, spatial variability contributes substantially more to the total variability in PM_2.5_ concentration in the non-AOD data set. In these conditions, meteorologic parameters in stage 1 have less predicting power in the non-AOD model than in the AOD model, and land use variables are more effective predictors in stage 2 of the non-AOD model. Although AOD is a highly significant predictor in the AOD model, it does not substantially improve model performance. This might be attributed partly to the high noise level of GASP AOD caused by simplistic aerosol model assumptions and errors in estimating surface reflectances (Kondragunta et al. 2008).

Because cloud cover and high surface reflectance are the major reasons why AOD data are missing, the difference in spatial and temporal variation of PM_2.5_ concentrations between the two models may be explained by stronger solar radiation and low surface reflectance facilitating more active vertical and horizontal mixing in the boundary layer, resulting in a spatially more smooth PM_2.5_ distribution. Higher temperature and direct sunlight also accelerate photochemical reactions to produce secondary PM species, such as sulfate, in the atmosphere. As a result, weather conditions are more effective predictors of PM_2.5_ concentrations in the AOD model. On the other hand, emissions of primary PM_2.5_ are more closely related to the distribution and profiles of sources than are meteorologic conditions. This is clearly shown in Figure 5, where although both models predicted elevated pollution levels at densely populated urban areas and also along major interstate highways, high concentrations are more dispersed in AOD model predictions than in non-AOD model predictions. In conclusion, GASP AOD can serve as a summary indicator of a set of weather conditions and land use types that stratify PM_2.5_ measurements into two distinct spatial patterns, and this differentiation has not been previously documented. Figures 5 and 6 suggest that even if land use regression models do not include AOD as a predictor variable, two separate models should be fitted to account for different PM_2.5_ spatial patterns related to AOD availability. Several significant improvements have been implemented in the latest GASP AOD retrieval after the completion of the present analysis (Kondragunta S, personal communication). These changes, including a refined azimuth angle definition, improved surface reflectance estimation method, and improved standard deviation calculation, may help reduce the noise level in GASP AOD data and therefore enhance its predicting power in our models.

The main limitation of the present study regarding PM_2.5_ prediction is that the two-stage GAMs are unable to account for the changing PM_2.5_ spatial patterns with time, which leads to some residual spatial autocorrelation. A full-scale space–time model with a space–time covariance model (i.e., space–time kriging) could account for changing spatial surfaces from day to day. Our work has focused on the impact of AOD on predicting PM_2.5_, but a full space–time model would be needed to better estimate daily PM_2.5_. Given our small domain, which includes only 32 monitoring sites, we have limited ability to estimate the effect of a large number of land use covariates such as distance to major roads and locations of large point sources, which can help in estimating spatial heterogeneity (Yanosky et al. 2008). Finally, model performance evaluated against monitoring data is always affected by the issue of comparing areal predictions by the model with point measurements. As illustrated in Georgopoulos et al. (2005), local-scale air quality models may be introduced to account for subgrid characteristics of photochemical reactions and dispersion.

## Figures and Tables

**Table 1:** Comparison of model fitting results for the AOD and the non-AOD models.

Measure	AOD	Non-AOD
Sample size (site days)	2,570	7,009
Significant predictors in stage 1	DOY, AOD, PBL, RH, TEMP, (*U*,*V*), SSC	DOY, AOD, PBL, RH, TEMP, (*U*,*V*), SSC
Stage 1 adjusted *R*^2^	0.77	0.43
Significant predictors in stage 2	AOD, POP, (*x*,*y*), CLASS_3	POP, CLASS_1, CLASS_3
Stage 2 adjusted *R*^2^	0.73	0.81
Model *R*^2^	0.79	0.48
CV *R*^2^	0.78	0.46
Mean prediction error	0.03 μg/m^3^	0.07 μg/m^3^
RMSPE	3.6 μg/m^3^	5.0 μg/m^3^

Abbreviations: DOY, day of year; POP, population density within each grid cell.
